# FOOT ECZEMA: THE ROLE OF PATCH TEST IN DETERMINING THE CAUSATIVE AGENT USING STANDARD SERIES

**DOI:** 10.4103/0019-5154.41649

**Published:** 2008

**Authors:** K S Priya, Ganesh Kamath, Jacintha Martis, Sukumar D, Narendra J Shetty, Ramesh M Bhat, B Nanda Kishore

**Affiliations:** *From Fr. Muller Medical College Hospital, Kankanady, Mangalore - 575 002, Karnataka, India*

**Keywords:** *Foot eczema*, *patch test*, *shoe dermatitis*

## Abstract

Foot dermatitis refers to the predominant involvement of feet in the eczematous process. This study is undertaken to determine the clinical pattern and causative agent in foot eczema and to evaluate the role of patch testing in determining the causative agent of foot eczema. Data was collected from 50 patients with foot eczema, who attended the out-patient department. The patch test was performed using Indian standard series. Patch test was positive in 88% of the patients. The most common site affected was the dorsal aspect of the foot (48%) and scaly plaque was the predominant morphological pattern. The highest number of patients (24%) showed positive reactions to mercaptobenzothiazole (MBT) and the lowest (4%) to neomycin sulfate. Rubber and rubber chemicals have been reported worldwide to be the most common sensitizer causing foot eczema. Thus, patch test has a major role in finding out the cause of foot eczema.

## Introduction

Foot dermatitis is one of the common problems observed by a dermatologist. It refers to predominant involvement of feet in the eczematous process. Patients suffering from this not only seek a solution for their problem but also demand to know the cause. Foot wear can be implicated as a cause for the eczema only if the eczematous process is restricted to the pattern of the foot wear. Hence, this study was undertaken to evaluate the role of patch test in finding out the cause for foot eczema.^1^ If the reason is found out, the clinician will obtain a distinct advantage in the subsequent management of the patient. An attempt is also made to correlate the clinical pattern of foot eczema with its cause.

## Materials and Methods

This study was conducted over a period of 16 months from November 2003 to February 2005. During this period, a total number of 50 patients with foot eczema, attending the out-patient department, were included in this study. Patient with eczematous lesions over the feet were interrogated for a detailed history with particular emphasis on age of onset, site of initial lesion, extent of dermatitis, seasonal variation, aggravating factors and association with atopy. Associated symptoms such as pain, pruritus, dryness, scaling, redness and oozing were also observed. Thorough clinical examination was carried out to know the distribution such as dorsal aspect, plantar aspect, fore foot, heel and instep. Skin scrapings for fungus were done to exclude fungal infections in all the doubtful cases before performing the patch test.

All the patients with foot eczema were patch tested using Indian standard series approved by the Contact and Occupational Dermatoses Forum of India (CODFI).

## Results

A total number of 50 patients completed the study, from which 32 (64%) were females and 18 (36%) males (Table 1). The peak age of onset was found to be between 20-30 years, which constituted 48%. The most common site to be initially involved was the dorsal aspect of the feet (48%), and dry scaly plaques was the common presentation.

**Table 1:** Age and sex

Age in years	Sex		
	
	Female	Male	Total
10-20	9	5	14
20-30	14	10	24
30-40	4	2	6
40-50	4	1	5
50-60	1	0	1
Total	32	18	50

Among various aggravating factors, 64% of the patients showed aggravation with footwear followed by medication, detergents, cement and plant (Table 2). Dermatitis was bilateral in 90% of the patients. Patch test was positive in 44 (88%) patients. Pruritus was the most common symptom that was seen in 88% of the patients, followed by pain, dryness, scaling and oozing. In the present study, 48% of the patients gave a history of deterioration of the lesions during winter.

**Table 2:** Aggravating factors

Aggravating factors	No. of patients
Footwear or socks	32
Medication	8
Detergents	6
Cement	3
Plant	1
Total	50

The highest number of patients (36%) showed positive reaction to mercaptobenzothiazole, followed by colophony, 4-phenylenediamine base (20%), potassium dichromate, formaldehyde, nickel sulfate, black rubber mix and thiuram mix. Among topical medications, neomycin sulfate constituted 4% and gentamycin constituted 12% (Table 3).

**Table 3:** Patch test report with Indian standard series

Antigen	No. of patients	±	+	++	+++
Potassium dichromate	6	1	3	2	-
Neomycin sulfate	2	-	2	-	-
4-phenylenediamine base	10	2	2	5	1
Nickel sulfate	3	1	2	-	-
Colophony	16	-	13	3	-
Gentamycin	6	2	4	-	-
Mercaptobenzothiazole	18	1	14	3	-
Black rubber mix	2	1	1	-	-
Thiuram mix	4	1	3	-	-
Formaldehyde	9	2	4	3	-

± - doubtful reaction; + - weakly positive reaction; ++ - strongly positive reaction; +++ - highly positive reaction

## Discussion

The most common age group of presentation of foot eczema in our study was between 20-30 years, which is in accordance with most studies.^2^ This is possibly because of the fact that this age group is the active phase of one's life where chances of exposure to allergens are more.

A total number of 50 patients were included in our study, from which 32% were females and 18% were males. This may be attributed to the fact that women come in contact with irritants and water and also use a variety of footwear more than men.

The most common site involved was on the dorsum of the feet corresponding to the shape of the foot wear (V-shaped chappals). This type of foot wear is usually worn without socks and is preferred by people living in warm and humid tropical climates as in this coastal town. Similar observations have been made in other studies.^3^ Several authors have reported that rubber-based adhesives that are universally used in the shoe industry are apparently the cause of shoe dermatitis.^4^,^5^ Soaps and detergents have been implicated as predisposing factors in various studies.^6^ The greatest hazard occurs in building and constructions sites.^7^ In our study, 88% of the patients gave a positive patch test result, from which MBT was the commonest sensitizer (36%); this observation was consistent with findings of other studies.^8^

## Conclusion

Foot eczemas are one among the many common dermatological disorders that are observed in a dermatology out-patients department. The most common morphological pattern is dry scaly plaques type. Patch testing has a major role to play in finding out the cause, and footwear is the main cause as all our patients who were tested positive for patch test showed positivity to one of the various compounds that is found in foot wear, MBT being the most common among them.

## References

[1] Rycroft RJ (1990). Is patch testing necessary?. Recent advances in dermatology.

[2] Handa S, Sharma SC, Sharma VK, Kaur S (1991). Foot wear dermatitis: Clinical patterns and contact allergens. Indian J Dermatol Venereol Leprol.

[3] Lyon CC, Tucker S, Karlberg AT, Beck MH (1999). Footwear dermatitis to colophony. Br J Dermatol.

[4] Romaguera C (1987). Shoe contact Dermatitis. Int J Dermatol.

[5] Bajaj AK, Gupta SC, Chatterjee AK, Singh KG (1988). Shoe dermatitis in India. Contact Dermatitis.

[6] Magnussan B, Hersle K (1965). Patch test methods: II, Regional variations of Patch test responses. Acta Derm Venerol.

[7] Calnan CD (1960). Cement dermatitis. J Occup Med.

[8] Blank IH, Miller OG (1952). A study of Rubber adhesives in shoes as the cause of dermatitis of the feet. JAMA.

